# Supplementary material for: Cross Talk between GlAQP and NOX Modulates the Effects of ROS Balance on Ganoderic Acid Biosynthesis of Ganoderma lucidum under Water Stress
Source: Microbiol Spectr. 2022 Nov 2;10(6):e01297-22. doi: 10.1128/spectrum.01297-22 (PMC9784773; doi:10.1128/spectrum.01297-22)
Supplement: Supplemental file 1 — Supplemental material. Download spectrum.01297-22-s0001.pdf, PDF file, 2.4 MB [file spectrum.01297-22-s0001.pdf]

Cross Talk between GlAQP and NOX Modulate Effects of ROS Balance on Ganoderic Acid Biosynthesis of *Ganoderma lucidum* under Water Stress

Cross Talk between AQP and NOX Regulates GA synthesis

Quanyu Zhu[a], Ang Ren[a,b], Juan Ding[a], Jian He[a], Mingwen Zhao[a], Qin He[a,b,#]

[a]Department of Microbiology, Key Laboratory of Agricultural Environmental Microbiology, Ministry of Agriculture, College of Life Sciences, Nanjing Agricultural University, Nanjing 210095, Jiangsu, P. R. China

[b]Sanya Institute of Nanjing Agricultural University, Sanya 572025, Hainan, P. R. China

The word counts for the abstract is 250 and the word count for the text is 4768.

[#] Corresponding author. Tel.: +86-25-84395326. Fax: +86-25-84395326.

E-mail: qhe@njau.edu.cn (Q. He).

Table S1. The primers used in this study

Primer	Sequence (5' to 3')	Description
GlAQP-F	ATGTCGACGCGTTCTATCAG	Get the full length of *GlAQP* gene
GlAQP-R	TTAGCCATTCAGGGGCGG	
RT-*GlAQP*-F	ATCGGTGCTTCGTTGG	Detects the *GlAQP* expression
RT-*GlAQP*-R	GGCGGCGTGACAGGAGTGG	
*GlAQP*i-F	TCGCCCACTACACAAAA	Get the silencing fragment of *GlAQP*
*GlAQP*i-R	ACCGAAGTCATATACGGCAG	
OE::*GlAQP*-F	CGGGATCCATGTCGACGCGTTCTATCAG	Construct overexpression vector of *GlAQP*
OE::*GlAQP*-F	GCTCTAGATTAGCCATTCAGGGGCGG	
RT-GL25914-F	TCGCAAAAGAGAAGCAT	Detects the AQP gene *GL25914-R1* expression
RT-GL25914-R	GGTCCCAACCAGTAGAT	
RT-GL22962-F	AGAAACAAACAGAGGC	Detects the AQP gene *GL22962-R1* expression
RT-GL22962-R	TGAATACTGGAGCACC	
RT-GL25783-F	ATGAGGAGCACAGCCAA	Detects the AQP gene *GL25783-R1* expression
RT-GL25783-R	CGTAGAACCCAAACCGA	
RT-GL25673-F	TACCTCCGATCCCGA	Detects the AQP gene *GL25673-R1* expression
RT-GL25673-R	CGAAGTGCTCCCTGC	
RT-18S-F	TATCGAGTTCTGACTGGGTTGT	Detects the 18S expression
RT-18S-R	ATCCGTTGCTGAAAGTTGTAT	
RT-*hmgr*-F	GTCATCCTCCTATGCCAAAC	Detects the *hmgr* expression
RT-*hmgr*-R	GGGCGTAGTCGTAGTCCTTC	
RT-*sqs*-F	CTGCTTATTCTACCTGGTGCTACG	Detects the *sqs* expression
RT-*sqs*-R	GGCTTCACGGCGAGTTTGT	
RT-*osc*-F	AGGGAGAACCCGAAGCATT	
RT-*osc*-F	CGTCCACAGCGTCGCATAAC	Detects the *osc* expression
RT-*gpx*-R	GGAAGCCGAGAATGACGAAG	
RT-*NOXA*-F	CGTAGAGTTCTTCTGGGTTTGC	Detects the *NOXA* expression
RT-*NOXA*-R	CTGCGTGAGGTAGATGTTGATA	
RT-*NOXB*-F	TTCGCGTCGATCCTCAAGT	Detects the *NOXB* expression
RT-*NOXB*-R	GTTCTGCGTGTCCTGCTCC	
RT-*NOXR*-F	GCAGCTATGGAGGCAGCATG	Detects the *NOXR* expression
RT-*NOXR*-R	TCGCGGTAGTACAGCTTCACG	

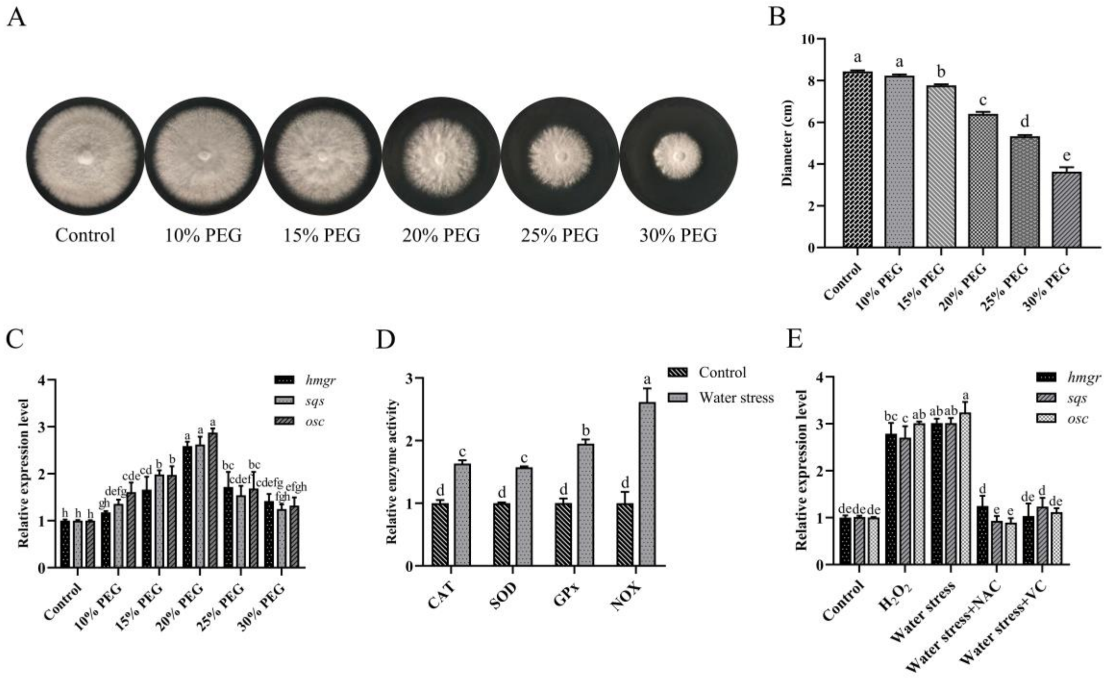

Fig. S1 Effect of water stress on the growth of *G. lucidum*, antioxidant enzyme activity and expression level of key enzyme genes in the GA biosynthetic pathway. (A) Image of the WT grown on CYM solid medium containing different concentrations of PEG at 28°C for 7 days. (B) The diameter of the colony of the WT grown on the CYM solid medium containing different concentrations of PEG at 28°C for 7 days. (C) The expression level of key enzyme genes (*hmgr*, *sqs* and *osc*) in the GA biosynthetic pathway. (D) The activity of CAT, SOD, GPx and NOX under water stress. (E) The expression level of key enzyme genes (*hmgr*, *sqs* and *osc*) in the GA biosynthetic pathway in WT treated with ROS scavengers under water stress. The values indicate the mean ± SD of three independent experiments. Different letters indicate significant differences between treatments (Duncan's multiple range test, $p < 0.05$).

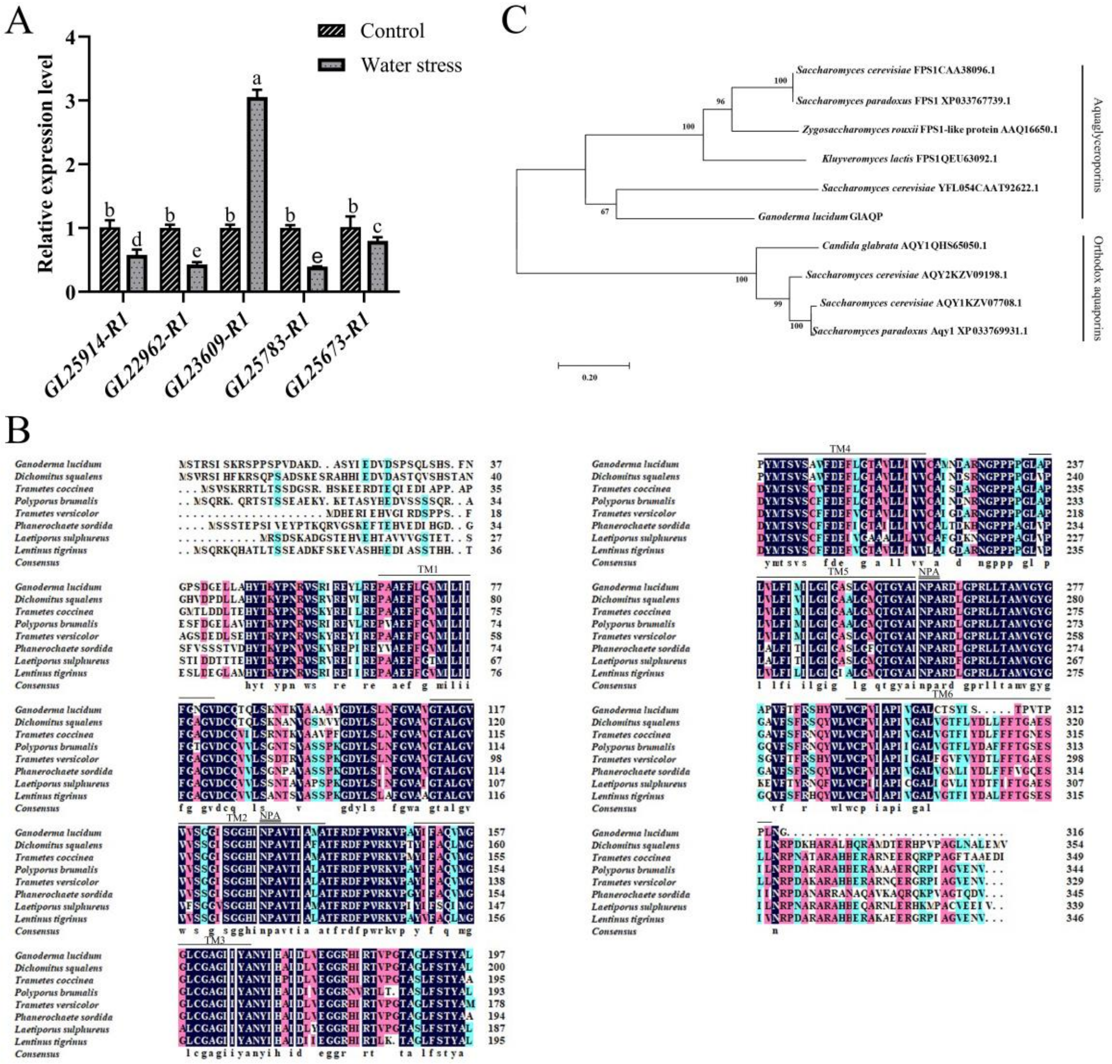

Fig. S2 The expression level of AQP genes, and the amino acid sequence comparison and phylogenetic analysis of GlAQP protein. (A) The expression level of AQP genes in WT under water stress. (B) The amino acid sequence of GlAQP protein is compared with the AQP amino acid sequence of other species using DNAMAN. The six transmembrane helices are marked with a single line and denoted as TM1-TM6, and the two conserved asparagine-proline-alanine (NPA) motifs are marked with a double line. The name and accession number of the species are: *Dichomitus squalens* (TBU23203.1), *Trametes coccinea* (OSD07493.1), *Polyporus brumalis* (RDX50348.1), *Trametes versicolor* (XP_008037753.1), *Phanerochaete sordida* (GJE99849.1),

Laetiporus sulphureus (XP_040766974.1), and *Lentinus tigrinus* (RPD62715.1). (C) The phylogenetic analysis of MEGA7 adopts the neighbor joining method, and the guiding consensus tree deduced from 1000 repeats represents the evolutionary history. The name and GenBank accession number are shown after the species name. The values indicate the mean ± SD of three independent experiments. Different letters indicate significant differences between treatments (Duncan's multiple range test, $p < 0.05$).

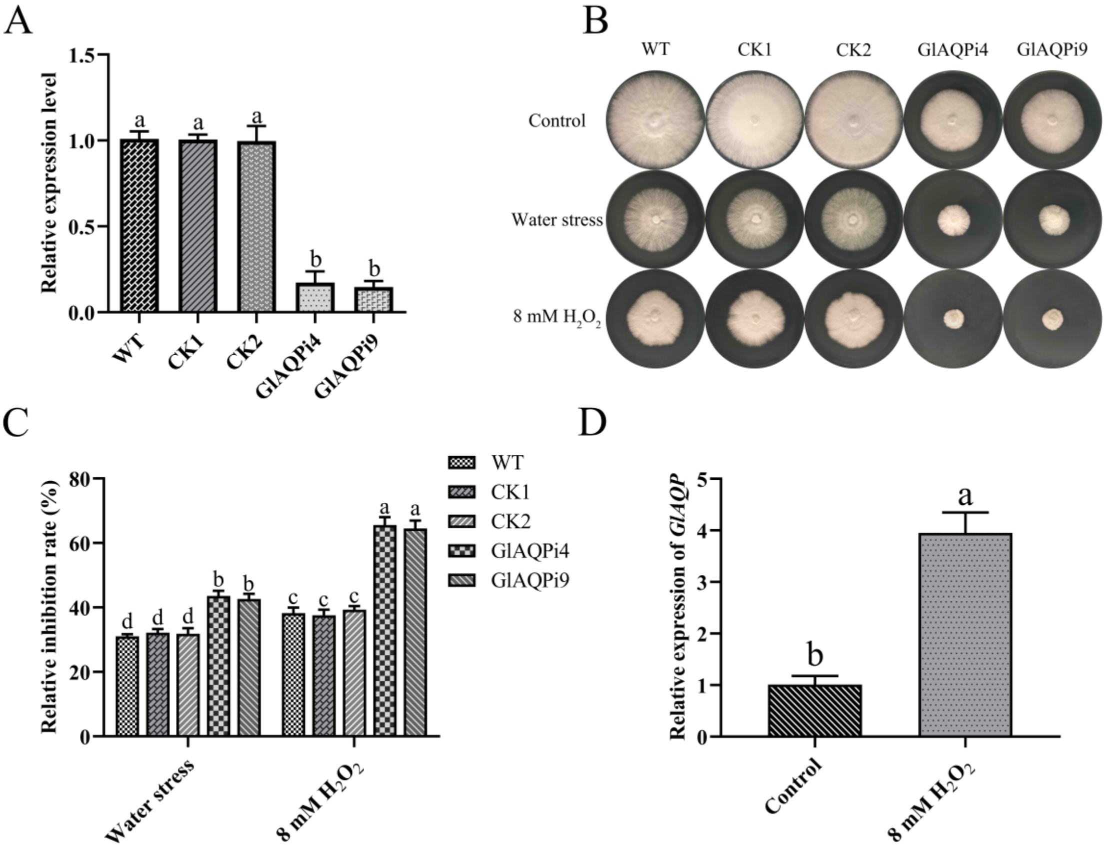

Fig. S3 Characterization of *GlAQP* silenced strains and their sensitivity to water stress and oxidative stress. (A) The expression level of *GlAQP* in *GlAQP* silenced strains. (B) Images of WT, CK, and *GlAQP* silenced strains grown at 28°C for 7 days. (C) Growth inhibition rate of *GlAQP* silenced strains on CYM solid medium containing 20% PEG or 8 mM H_2O_2. Growth inhibition rate = 100% × [colony diameter (untreated condition) - colony diameter (stress condition)] / colony diameter (untreated condition). (D) The expression level of *GlAQP* in WT under oxidative stress. The values indicate the mean ± SD of three independent experiments. Different letters indicate significant differences between treatments (Duncan's multiple range test, $p < 0.05$; Student's t-test: $p < 0.05$).

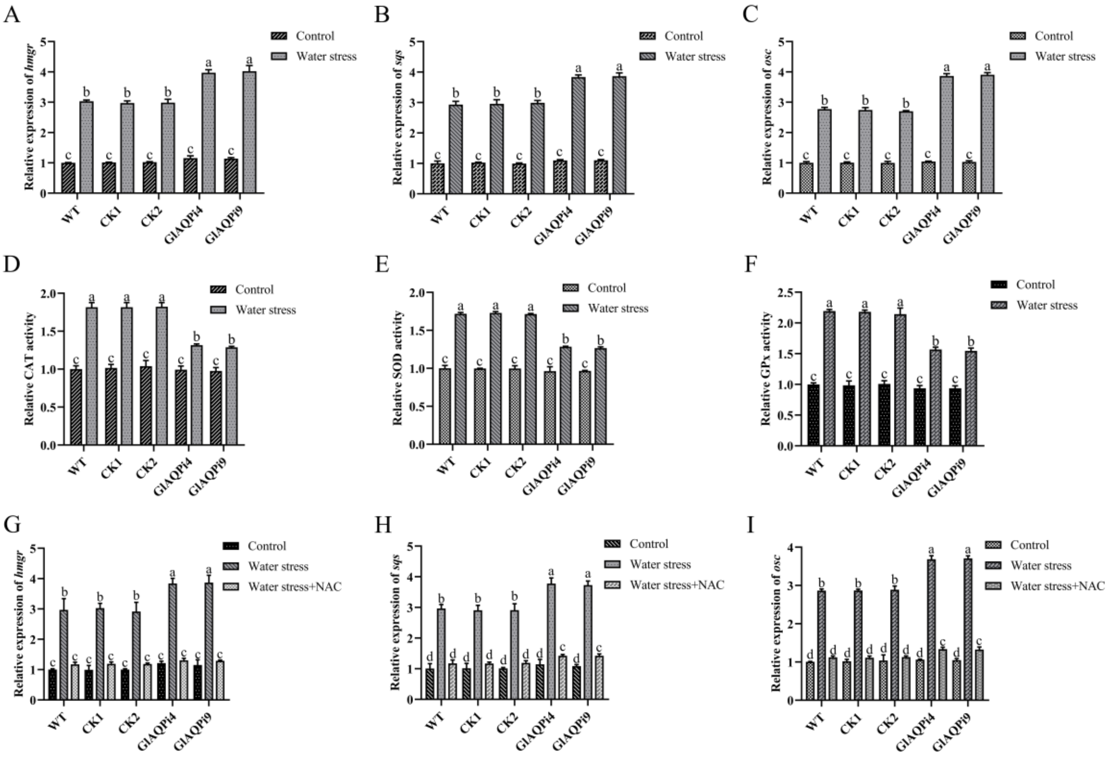

Fig. S4 The expression level of key enzyme genes in GA biosynthesis pathway and the activity of antioxidant enzymes in *GlAQP* silenced strains. The expression level of key enzyme genes, *hmgr* (A), *sqs* (B) and *osc* (C) in the GA biosynthetic pathway in *GlAQP* silenced strains under water stress. The activity of CAT (D), SOD (E) and GPx (F) in *GlAQP* silenced strains under water stress. The expression level of key enzyme genes, *hmgr* (G), *sqs* (H) and *osc* (I) in the GA biosynthetic pathway in *GlAQP* silenced strains treated with 0.5 mM ROS scavenger NAC under water stress. The values indicate the mean ± SD of three independent experiments. Different letters indicate significant differences between treatments (Duncan's multiple range test, $p < 0.05$).

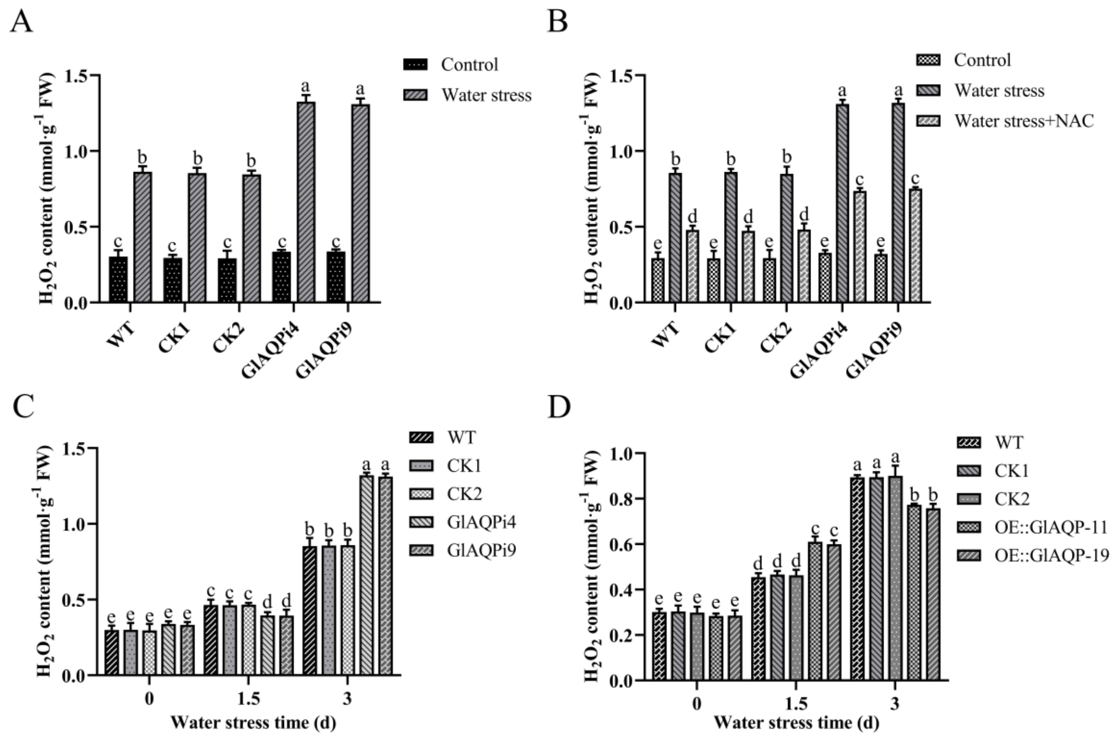

Fig. S5 H_2O_2 content in *GlAQP* silenced strains and *GlAQP* overexpressing strains under water stress. (A) H_2O_2 content in *GlAQP* silenced strains under water stress. (B) H_2O_2 content in *GlAQP* silenced strains treated with 0.5 mM NAC under water stress. (C) H_2O_2 content in WT, CK and *GlAQP* silenced strains of *G. lucidum* under water stress at early and late stages. (C) H_2O_2 content in WT, CK and *GlAQP* overexpressing strains of *G. lucidum* under water stress at early and late stages. The values indicate the mean ± SD of three independent experiments. Different letters indicate significant differences between treatments (Duncan's multiple range test, $p < 0.05$).

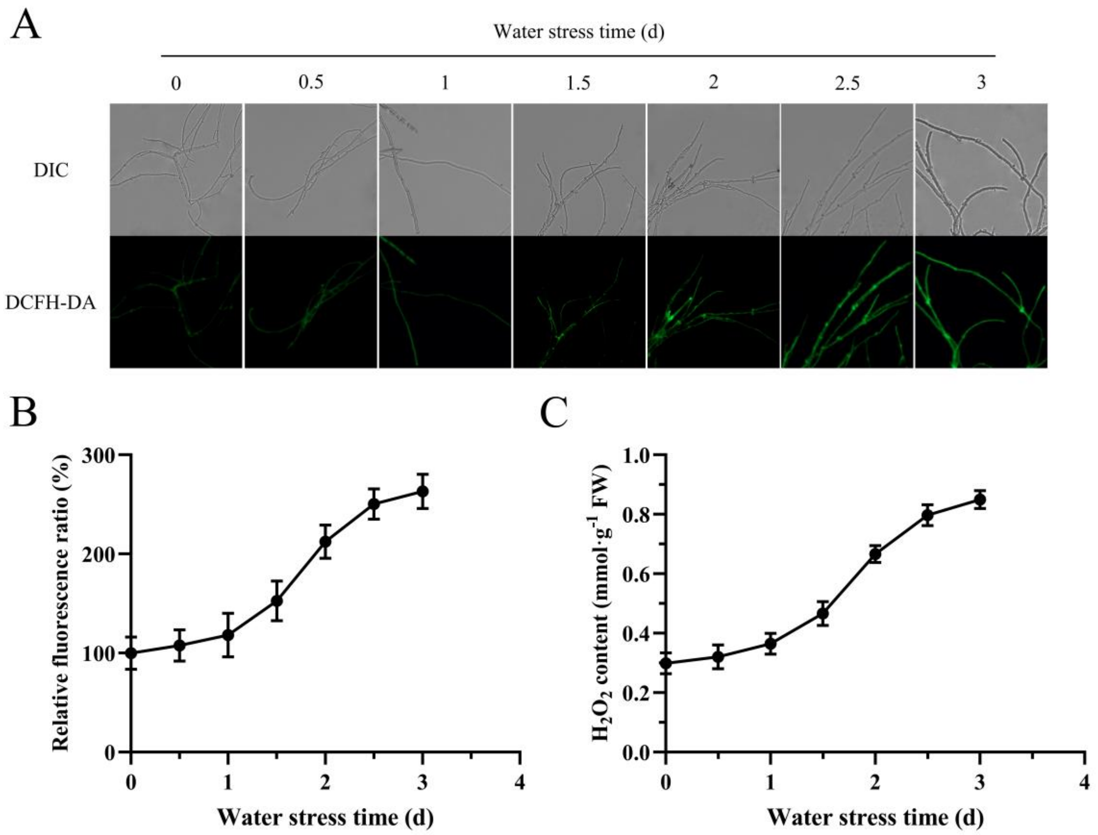

Fig. S6 Change of ROS level in WT during the whole water stress process. (A) Change in ROS level detected by DCFH-DA staining in WT during the whole water stress process. (B) Change in ROS fluorescence ratio in WT during the whole water stress process. (C) H_2O_2 content in WT during the whole water stress process. The values indicate the mean ± SD of three independent experiments.

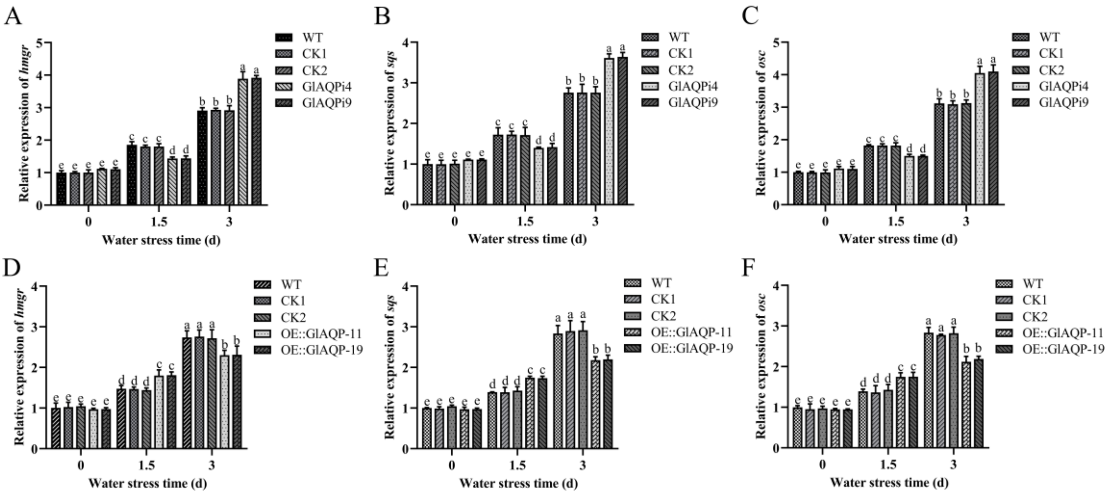

Fig. S7 The expression level of key enzyme genes in GA biosynthesis pathway in *GlAQP* silenced and overexpressing strains under water stress at early and late stages. The expression level of *hmgr* (A), *sqs* (B) and *osc* (C) in WT, CK and *GlAQP* silenced strains under water stress at early and late stages. The expression level of *hmgr* (D)*, sqs* (E) and *osc* (F) in WT, CK and *GlAQP* overexpressing strains under water stress at early and late stages. The values indicate the mean ± SD of three independent experiments. Different letters indicate significant differences between treatments (Duncan's multiple range test, $p < 0.05$).

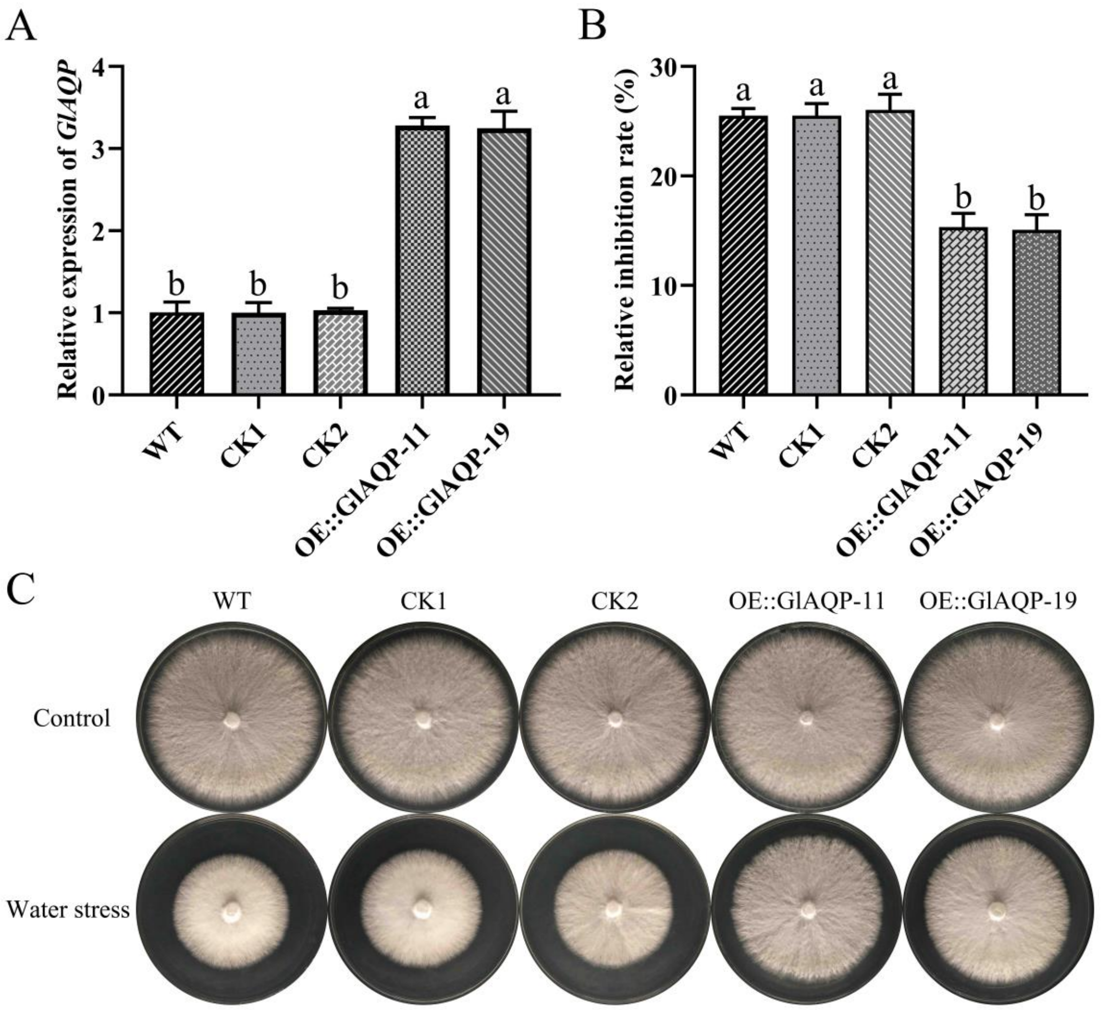

Fig. S8 Characterization of *GlAQP* overexpressing strains and their sensitivity to water stress. (A) The expression level of *GlAQP* in WT, CK and *GlAQP* overexpressing strains. (B) Growth inhibition rate of *GlAQP* silenced strains on CYM solid medium containing 20% PEG. (C) Images of WT, CK, and *GlAQP* overexpressing strains grown at 28°C for 7 days. The values indicate the mean ± SD of three independent experiments. Different letters indicate significant differences between treatments (Duncan's multiple range test, $p < 0.05$).

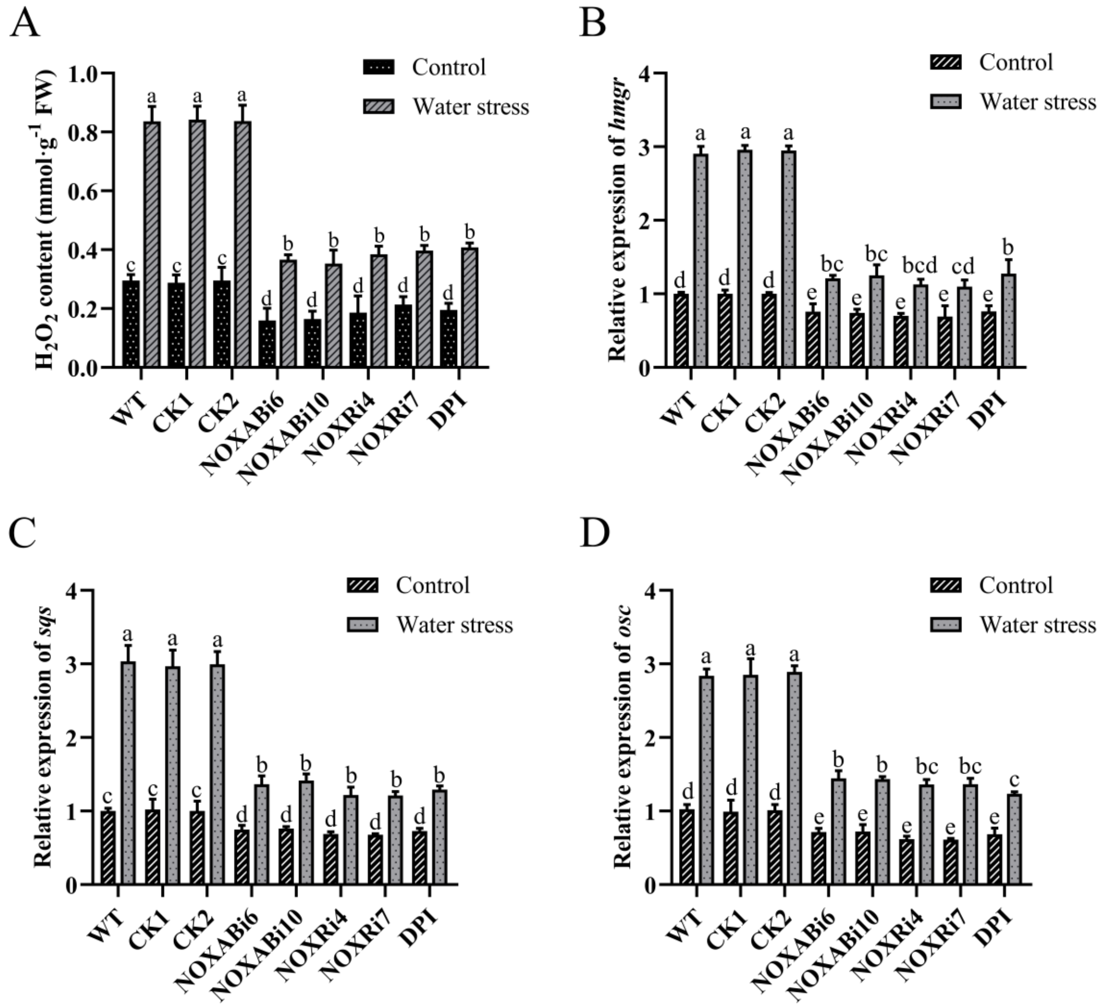

Fig. S9 H_2O_2 content and the expression level of key enzyme genes in the GA biosynthesis pathway in *NOX* silenced strains and DPI-treated WT under water stress. (A) H_2O_2 content in *NOX* silenced strains and 10 μM DPI-treated WT under water stress. The expression level of *hmgr* (B)*,* *sqs* (C) and *osc* (D) in *NOX* silenced strains and 10 μM DPI-treated WT under water stress. The values indicate the mean ± SD of three independent experiments. Different letters indicate significant differences between treatments (Duncan's multiple range test, $p < 0.05$).

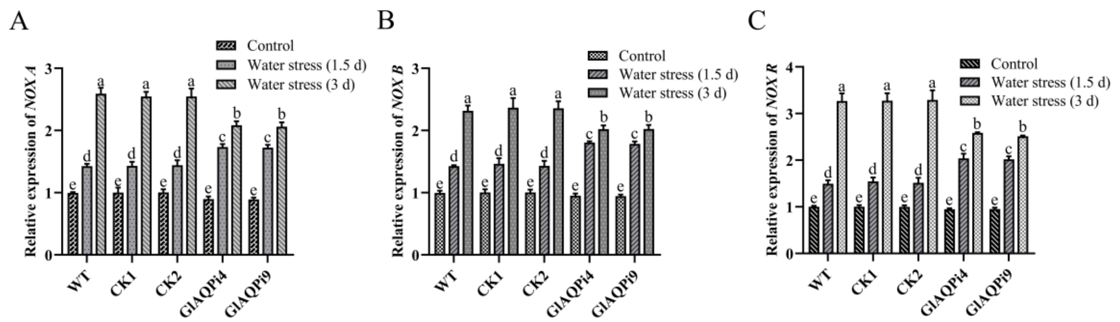

Fig. S10 The transcription level of *NOXA*, *NOXB* and *NOXR* in *GlAQP* silenced strains under water stress. Transcription level of *NOXA* (A), *NOXB* (B) and *NOXR* (C) in WT, CK and *GlAQP* silenced strains of *G. lucidum* under water stress at early and late stages.
